# How large are the nonspecific effects of acupuncture? A meta-analysis of randomized controlled trials

**DOI:** 10.1186/1741-7015-8-75

**Published:** 2010-11-23

**Authors:** Klaus Linde, Karin Niemann, Antonius Schneider, Karin Meissner

**Affiliations:** 1 Institute of General Practice, Technische Universität München, Orleansstrasse 47, D-81667 Munich, Germany; 2 Institute of Medical Psychology, Ludwig-Maximilians-University, Goethestrasse 31, D-80336 Munich, Germany

## Abstract

**Background:**

While several recent large randomized trials found clinically relevant effects of acupuncture over no treatment or routine care, blinded trials comparing acupuncture to sham interventions often reported only minor or no differences. This raises the question whether (sham) acupuncture is associated with particularly potent nonspecific effects. We aimed to investigate the size of nonspecific effects associated with acupuncture interventions.

**Methods:**

MEDLINE, Embase, Cochrane Central Register of Controlled Clinical Trials and reference lists were searched up to April 2010 to identify randomized trials of acupuncture for any condition, including both sham and no acupuncture control groups. Data were extracted by one reviewer and verified by a second. Pooled standardized mean differences were calculated using a random effects model with the inverse variance method.

**Results:**

Thirty-seven trials with a total of 5754 patients met the inclusion criteria. The included studies varied strongly regarding patients, interventions, outcome measures, methodological quality and effect sizes reported. Among the 32 trials reporting a continuous outcome measure, the random effects standardized mean difference between sham acupuncture and no acupuncture groups was -0.45 (95% confidence interval, -0.57, -0.34; I^2 ^= 54%; Egger's test for funnel plot asymmetry, *P *= 0.25). Trials with larger effects of sham over no acupuncture reported smaller effects of acupuncture over sham intervention than trials with smaller nonspecific effects (β = -0.39, *P *= 0.029).

**Conclusions:**

Sham acupuncture interventions are often associated with moderately large nonspecific effects which could make it difficult to detect small additional specific effects. Compared to inert placebo interventions, effects associated with sham acupuncture might be larger, which would have considerable implications for the design and interpretation of clinical trials.

## Background

In recent years, there has been increasing evidence from large randomized trials and systematic reviews showing that patients receiving acupuncture report better outcomes than patients receiving no treatment or usual care only (for example, [1,2]). A large trial on low back pain [3] and a meta-analysis of migraine trials [4] even found superiority over guideline-oriented conventional care. At the same time, many recent high-quality trials comparing true acupuncture with a sham acupuncture intervention found only minor or even no differences (see [4-7] for systematic reviews). The interpretation of this evidence is controversial. Some authors argue that the better effects over no treatment and usual care are only due to the usual placebo effects and bias [8]. Some authors argue that most sham acupuncture interventions are physiologically active [9,10], and others contend that sham acupuncture interventions might be associated with particularly potent nonspecific or placebo effects [11,12].

Treatment effects are considered specific if they are attributable solely, according to the theory of the mechanism of action, to the characteristic component of an intervention [13,14]. Effects which are associated with the incidental elements of an intervention are considered nonspecific effects (synonymous with placebo effects). Nonspecific effects are mostly thought to be due to psychobiological processes triggered by the overall therapeutic context [15]. They have to be distinguished from the natural course of disease, regression to the mean, effects of being in a study, cointerventions and, as far as possible, from reporting and other biases [16,17]. The total effect of an intervention consists of both specific and nonspecific effects [18].

Separating characteristic and incidental elements of an intervention is straightforward in pharmacology, but is difficult in other interventions such as psychotherapy [19]. Acupuncture involves the insertion and manipulation of needles into defined points of the body. While a variety of mechanistic models exist, the exact mechanism of action is unclear [20]. This makes it difficult to devise a placebo intervention which is both inert and indistinguishable and reliably separates specific and nonspecific effects. The frequent use of the term *sham intervention *instead of *placebo *partly reflects this problem. Sham interventions in clinical trials of acupuncture typically vary from "true" acupuncture in one or both of the following aspects [21]: location of points (for example, stimulation of nonindicated points or outside known points) and skin penetration (for example, use of fixed telescope "placebo" needles with a blunt tip). If some or most of these sham interventions should indeed be physiologically active, such trials would not compare acupuncture to a placebo but to an active intervention, making it more difficult to detect significant differences.

This problem would also apply if (sham) acupuncture would be associated with more potent placebo effects than other interventions. Both invasive and noninvasive sham acupuncture interventions exert (like true acupuncture) mild painful stimuli. It has been hypothesized that such interventions might trigger enhanced placebo effects by simultaneously acting on sensory, cognitive and emotional levels [12]. There is also evidence that the same sham acupuncture intervention can have quite different effects when provided in different contexts [22]. Placebo research indicates that in many situations, the therapeutic context associated with an intervention matters more than the placebo intervention itself [15]. The therapeutic context depends not only on the specific therapeutic ritual applied but also on experiences, attitudes and preferences of patients and providers, the patient-provider interaction, the setting and the cultural background [11]. Given the positive attitudes and expectation toward complementary therapies, it seems possible that complex rituals such as acupuncture could provoke significant psychobiological responses.

The most straightforward way to investigate whether sham acupuncture is associated with larger effects than a pharmacological placebo would be in randomized trials including both these interventions. The only trial using such an approach indeed found a significant superiority of sham acupuncture [23]. Another, albeit methodologically weaker, possibility is to compare differences between sham acupuncture interventions and no-treatment control groups in acupuncture trials with those of (other) placebos and no-treatment control groups in other trials. Hróbjartsson and Gøtzsche [24-26] have repeatedly reviewed all available trials, including both a placebo or sham and a no-treatment group for any condition. The latest update of their Cochrane review includes a total of 234 trials. In a preplanned subgroup analysis, they found that studies using "physical placebos" (including sham acupuncture) reported larger placebo effects (standardized mean difference (SMD) -0.31; 95% confidence interval (CI) -0.41, -0.22) than studies using "pharmacological placebos" (SMD -0.10; 95% CI -0.20, -0.01) [26]. In a reanalysis of their data, we separated the trials in which the physical placebo was sham acupuncture from those which used other physical placebos. Effect sizes were significantly larger in trials using sham acupuncture than in trials using other physical placebos (SMDs -0.41 (-0.56, -0.24) vs -0.26 (-0.37, -0.15); *P *= 0.007) [27].

The Cochrane review [26] and our reanalysis of these data did not include a number of recent rigorous, large acupuncture trials which included both a sham group and a no-treatment group. Furthermore, these reviews did not investigate whether large nonspecific effects might make it difficult to detect specific effects. Therefore, we have performed a systematic review of acupuncture trials in any condition including both sham and no-treatment groups published through April 2010. Our primary aim was to investigate the size of nonspecific effects of acupuncture (difference between sham acupuncture vs no acupuncture). Our secondary aims were to investigate factors (such as type of sham intervention, condition, study quality or intensity of cointerventions) possibly influencing the size of such nonspecific effects and to quantify specific (difference acupuncture vs sham acupuncture) and total effects of acupuncture (difference acupuncture vs no acupuncture) in the included trials.

## Methods

### Selection criteria

To be included, studies had to meet the following criteria: (1) allocation to groups was explicitly randomized; (2) participants were persons treated for any illness or for preventative purposes; trials in healthy volunteers measuring physiological outcomes were excluded; (3) intervention involving the insertion of needles described as acupuncture at acupuncture points, pain or trigger points with or without stimulation; trials on interventions without skin penetration (for example, laser acupuncture) were excluded; (4) sham interventions described as sham, placebo, dummy or fake treatment which differed from true acupuncture in at least one of two key aspects (skin penetration or point location); (5) no-acupuncture control group had to be a second control group in which participants received neither true nor sham acupuncture; participants could be either completely untreated or receive treatments which were also administered in the true and sham acupuncture groups (for example, rescue medication, basic treatment or routine care); and (6) a clinical outcome for which the calculation of an effect size estimate was possible.

### Data sources and searches

To identify potentially relevant studies, we searched MEDLINE (from 1966 to April 2010) and Embase (from 1988 to April 2010) for all sham-controlled trials of acupuncture (see Additional file 1, Search strategies). Furthermore, we searched the Cochrane Central Register of Controlled Trials using a search strategy based on a Cochrane review of randomized trials with placebo and no-treatment controls in all medicine [25]. While Chinese trials identified by our search were eligible, we did not search specific Chinese databases. One reviewer screened titles and abstracts of all references identified and excluded those which were clearly irrelevant. Full texts of all remaining articles were obtained and assessed independently for eligibility by two reviewers. Disagreements or uncertainties were resolved by discussion.

### Data extraction and quality assessment

One reviewer extracted information on the following aspects from included studies using a standard form: diagnosis; recruitment; number and type of study centers; number and types of intervention and control groups; details on acupuncture and sham interventions; how patients were informed about these interventions; qualification of acupuncturists; cointerventions; study duration, number of patients randomized, analyzed and dropping out (per group); age; gender; results on the main outcome measures; important secondary outcomes and responder data. A second reviewer checked all extraction of study results against the original publications. Trials were considered to have lower risk of bias if they reported an adequate method of randomization concealment and had a dropout rate below 15% [28]. For our main analyses, we used the following strategy to choose the outcome: (1) it should be a continuous outcome (mean and standard deviation available, or the standard deviation could be calculated from standard errors or confidence intervals, for example; we did not impute standard deviations for studies without available data on variability or precision); (2) the timing should be as close as possible to the completion of treatment; (3) when there was a clearly predefined main outcome measure, we chose this measure (but always preferred the measurement at the end of treatment over other time points or change from baseline); (4) when there was no predefined single main outcome measure, two reviewers independently chose the outcome considered most important (two disagreements were resolved by discussion); (5) If available, we used intention to treat data; otherwise, we used the data as presented in the publication. If a trial had more than one intervention (for example, an individualized and a standardized intervention) or more than one sham group, the data were pooled. For more recent studies, we tried to contact authors to inquire for further information if data for meta-analysis were missing.

### Data synthesis and analysis

The Cochrane Collaboration's Review Manager RevMan 5 software was used for meta-analyses. Three comparisons were investigated: sham acupuncture versus no acupuncture (primary comparison), acupuncture versus sham acupuncture, and acupuncture versus no acupuncture. Studies were categorized into the clinical categories of chronic pain studies, short-term studies (that is, studies with an observation period of less than 3 days), and other studies.

The main analysis was based on trials reporting a continuous outcome measure using the standardized mean difference (SMDs; difference between the means/pooled standard deviation) as an effect size estimate. As we assumed that studies would be clinically heterogeneous, a random effects model with the inverse variance method was used for meta-analysis. Negative SMDs indicated a beneficial effect of sham acupuncture over no acupuncture, acupuncture over sham acupuncture and acupuncture over no acupuncture, respectively. SMDs ≤ -0.4 were considered small effects, those between -0.41 and -0.7 were considered moderate effects and those > -0.7 were considered large effects [29]. To investigate statistical heterogeneity, RevMan 5 uses Tau^2^, Chi^2 ^and I^2^. We considered I^2 ^values between 30% and 60% as indicating moderate heterogeneity and higher values as indicating substantial heterogeneity. Subgroup comparisons were performed using the method described by Deeks *et al. *[30] and implemented in RevMan 5. Egger's test was used to assess funnel plot asymmetry [31].

To check the robustness of results, we performed sensitivity analyses (1) including three-armed studies which had been excluded because they did not meet all inclusion criteria, but still could be considered because they addressed the questions investigated in this review ("borderline" studies; see Results); (2) using different outcomes for studies with more than one relevant outcome at the completion of treatment; and (3) using dichotomous outcome measures (with a relative risk <1 indicating a beneficial effect).

For exploratory analyses, we defined further subgroups: larger (at least 100 patients) and smaller (< 100 patients) comparisons; lower and higher risk of bias (see data extraction and quality assessment); studies with intense or less intense cointerventions in all study arms, with and without skin penetration (and depending on where needles were placed) in sham groups; studies with and without a clearly defined main outcome measure; and studies describing sham in the consent procedure as another treatment or placebo. In multivariate random effects meta-regression analyses, we investigated simultaneously the influence of risk of bias, cointerventions, skin penetration in the sham group and condition (chronic pain vs others). Analyses were carried out using the restricted information maximum likelihood (REML) method. For meta-regression analyses, PASW versions 17.0 and 18.0 software (SPSS, Chicago, IL, USA) using additional macros described by Wilson was used [32]. To investigate the hypothesis that there is an inverse correlation between specific and nonspecific effects (that is, trials with large nonspecific effects are less likely to find large specific effects than are trials with small nonspecific effects), we performed a linear regression analysis using the inverse of the squared pooled standard error as a weighting factor.

## Results

### Literature search and selection

**Figure 1:**
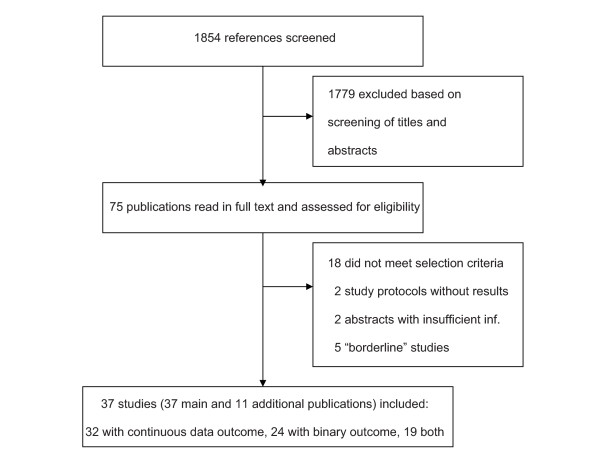
**Flow chart**.

The literature search identified a total of 1854 references, of which 1779 were excluded in the screening process as they clearly did not meet the inclusion criteria (see Figure 1). The full text of the remaining 75 references was formally assessed for eligibility. A total of 37 studies [33-69] met the inclusion criteria. Eleven additional publications reported protocols or treatment details of trials included in the review or reported the same results in another language (see Additional file 1, Table S1). Eighteen articles did not meet the inclusion criteria, and two were protocols of ongoing trials (see Additional file 1, Table S2). Two abstracts reported minimal information on probably eligible trials including results for a dichotomous outcome [70,71]; attempts to obtain further information from the authors were unsuccessful. In four other studies, patients in the no-acupuncture control group received minor interventions not provided in the other two groups [72-75]. Finally, for one study presenting an asymmetric confidence interval for the continuous main outcome measure, we were unable to unambiguously calculate the standard deviation [76]. The latter five trials were included in a sensitivity analysis as "borderline" studies.

### Description of included studies

The 37 eligible trials included a total of 5754 patients (median 75, minimum 30, and maximum 638). Fourteen trials (3369 patients) addressed chronic pain or a condition associated with chronic pain (Table 1); eight were short-term trials with a duration of less than 3 days (522 patients; Table 2) investigating whether acupuncture is helpful for sedation, anxiety, pain or nausea associated with surgical operations, endoscopic interventions or labor; and 15 trials (1863 patients) addressed a variety of other conditions (Table 3). Ten of the 14 chronic pain trials, but only six of the remaining 23 studies, reported an adequate method of allocation concealment. Dropout rates were between 54% and 95% in three addiction trials, but low in most other studies. Ten chronic pain trials and three trials of other conditions reported an adequate method of allocation concealment and a dropout rate below 15% and were classified as having a lower risk of bias.

**Table 1:** Characteristics of included trials: Chronic pain trials

Trial	Clinical problem	Sample size (% dropout rate)	Concealment	Outcome used for meta-analysis	Intervention details		Standard basic care in all groups
Birch & Jamison [38]	Chonic myofascial neck pain	46 (22%)	Unclear	Change from baseline on pain intensity rating scale	N:14 D:30 T:12w	Ac:C JA S:I C 2	NSAIDs if needed
Brinkhaus *et al. *[39]	Chronic low-back pain	298 (6%)	Adequate	PMOM: VAS pain intensity week 8	N:12 D:30 T:8w	Ac:B CA S:I B 1	NSAIDs if needed
Cherkin *et al. *[42]	Chronic low-back pain	638 (5%)	Adequate	PMOM: Roland Morris Disability Questionnaire week 8	N:10 D:20 T:7w	Ac1:A CA Ac2:C CA S:C II 3	Self-care book, usual care as needed
Facco^a ^*et al. *[44]	Migraine	160 (21%)	Adequate^b^	Migraine Disability Index (MIDAS) at 3 months	N:20 D:30 T:11w	Ac:A CA S1:II A 3 S2:II C 3	Rizatriptan for attacks in all patients
Foster *et al. *[46]	Osteoarthritis of the knee	352 (7%)	Adequate	WOMAC pain scale at 6 weeks (PMOM: 6 months)	N:6 D:30 T:3w	Ac:A CA S:II A 3	Individual exercise, advice, NSAIDs if needed^d^
Helms [49]	Primary dysmenorrhea	48 (10%)	Unclear	Monthly pain score week 12	N:9 D:30 T:12w	Ac:C CA S:I C 1	No treatment
Kotani *et al. *[51]	Treatment-resistant pain at abdominal scares	70 (0)	Adequate	VAS intensity continuous pain after treatment	N:20 D^c^ T:4w	Ac:A TA S:I A 1	Diclofenac as necessary
Lee & Lee [52]	Chronic prostatitis/chronic pelvic pain	39 (19%)	Unclear	PMOM: Change NIH-Chronic Prostatitis Symptom Index	N:12 D:20 T:6w	Ac:C EA S:I C 1	Advice and basic exercises
Leibing *et al. *[53]	Chronic low-back pain	131 (13%)	Unclear	Decrease intensity of pain (VAS) at 12 weeks	N:20 D:30 T:12w	Ac:C CA S:I C 1	26 sessions standardized physiotherapy^d^
Linde *et al. *[56]	Migraine	302 (9%)	Adequate	PMOM: Days with at least moderate headache in weeks 9 to 12	N:12 D:30 T:8w	Ac:B CA Sc:I B 1	Attack medication as needed
Melchart *et al. *[58]	Tension-type headache	270 (8)%	Adequate	PMOM: Number of days with headache in weeks 9 to 12	N:12 D:30 T:8w	Ac:B CA S:I B 1	Pain medication as needed
Molsberger *et al. *[59]	Chronic low-back pain	186 (6%)	Adequate	VAS pain intensity after 1 month (dichotomous PMOM)	N:12 D:30 T:4w	Ac:B CA S:I C 1	Orthopedic rehabilitation program^d^
Suarez-Almazor^a ^*et al. *[64]	Osteoarthritis of the knee	535 (8%)	Adequate^b^	WOMAC pain scale at 3 months	N:12 D:20 T:6w	Ac:C CA S:I C 1	NSAIDs and analgesics as before study
Witt *et al. *[67]	Osteoarthritis of the knee	294 (5%)	Adequate	PMOM: WOMAC total score after baseline at 8 weeks	N:12 D:30 T:8w	Ac:B CA S:I B 1	NSAIDs if needed

^a^Two sham groups with different context. ^b^Additional information received from author. ^c^Treatment with intradermal needles. ^d^Classified as intense cointervention likely to influence the outcome. PMOM, explicitly predefined (regarding *type and timing*) confirmatory main outcome measure; VAS, visual analogue scale; N, number of treatment sessions; D, duration of each treatment session; T, total duration of treatment in weeks; Ac, Acupuncture; S, sham intervention; A, individualized; B, semistandardized; C, standardized; CA, needling at classical acupuncture body points; EA, electroacupuncture (needles stimulated with electrical currency); EarA, ear acupuncture (needling at ear points); JA, Japanese acupuncture (superficial needling); TA, acupuncture at trigger points; I, penetrating; II, nonpenetrating; 1, needled outside known points; 2, acupuncture points not indicated for condition needled; 3, at correct points; NSAIDs, nonsteroidal anti-inflammatory drugs; WOMAC, Western Ontario and McMaster Universities Osteoarthritis Index.

**Table 2:** Characteristics of included trials: Short-term trialsa

Trial	Clinical problem	Sample size (% dropout)	Concealment	Outcome used for meta-analysis	Intervention details		Standard basic care in all groups
Cabrini *et al. *[41]	Bronchoscopy	48 (0)	Unclear	VAS discomfort after bronchoscopy	N:1 D:20 T:-	Ac:C CA S:I C 1	Lidocaine as needed
Dundee *et al. *[43]	Perioperative nausea (minor gynecologic operations)	75 (0)	Unclear	Number of patients vomiting	N:1 D:5 T:-	Ac:C CA S:I C 1	Premedication 10 mg nalbophine
Fanti *et al. *[45]	Colonoscopy	30 (0)	Unclear	Satisfaction with sedation using a verbal rating scale	N:1 D:4 T:-	Ac:C EA S:I C 1	Midazolam 0.02 mg/kg before and during colonoscopy^b^
Gioia *et al. *[48]	Sedation during cataract surgery	75 (0)	Unclear	Postoperative anxiety evaluation (VAS)	N:1 D:60 T:-	Ac:C CA S:I C 1	Topical eye anesthesia (lidocaine 4%)
Karst *et al. *[50]	Anxiety and tooth extraction	48 (0)	Unclear	VAS anxiety	N:1 D:25 T:-	Ac:C EarA S:II C 1	Local anesthesia (articaine hydrochloride)
Li *et al. *[55]	Colonoscopy	36 (0)	Unclear	VAS pain intensity	N:1 D:30 T:-	Ac:C EA S:I C 1	Routine analgesia and sedation as needed
Rusy *et al. *[62]	Postoperative nausea after tonsillectomy	120 (0)	Unclear	Incidence of nausea, vomiting, rescue therapy in first 24 hours	N:1 D:20 T:-	Ac:C EA S:I C 2	Standardized anesthesia, analgesia as needed
Ziaei & Hayipour [69]	Pain reduction and relaxation during labor	90 (unclear)	Unclear	VAS pain after 2 hours	N:1 D:n.i.. T:-	Ac:C CA S:I C 1	Unclear

^a^See Table 1 footnotes for definitions of abbreviations. ^b^Classified as intense cointervention likely to influence the outcome.

**Table 3:** Characteristics of included trials: Trials on various other conditionsa

Trial	Clinical problem	**Sample size (% dropout**)	Concealment	Outcome used for meta-analysis	Intervention details		Standard basic care in all groups
Allen *et al. *[33]	Depression	38 (11%)	Unclear	Hamilton Rating Scale for Depression after 8 weeks	N:12 D:n.i. T:8w	Ac:A CA S:I A 2	Probably no treatment at all
Allen *et al. *[34]	Depression	157 (13%)	Unclear	Hamilton Rating Scale for Depression after 8 weeks (PMOM)	N:12 D:20 T:8w	Ac:A CA S:I A 2	Probably no treatment at all
Asher *et al. *[35]	Induction of labor	89 (0)	Adequate	PMOM: Time to delivery	N:7 D:30 T:2w	Ac:C CA S:I A 1	Routine prenatal care
Aune *et al. *[36]	Recurrent urinary tract infections	67 (unclear)	Unclear	Patients without infection	N:8 D:20 T:4w	Ac:A CA S:I C 1	No treatment
Avis *et al. *[37]	Menopausal hot flashes	56 (0)	Adequate^b^	Reduction in hot flash frequency^b^	N:16 D:30 T:8w	Ac:B CA S:B A 2	Continuation of nondrug treatment used before trial
Bullock *et al. *[40]	Addiction (cocaine abuse)	236 (59%)	Unclear	Addiction severity scale drug use in week 8	N:28 D:45 T:8w	Ac:C EarA S:I C 2	Eden psychosocial programming^d^
Freire *et al. *[47]	Moderate obstructive sleep apnea syndrome	36 (28%)	Unclear	Apnea-hypopnea index after 12 weeks	N:10 D:30 T:10w	Ac:C CA S:I C 1	Offer to receive sleep hygiene counseling
Lembo^b ^*et al. *[54]	Irritable bowel syndrome (IBS)	231 (8%)	Adequate	PMOM: IBS Global Improvement Scale week 3	N:6 D:20 T:3w	Ac:B CA S:II B 1	Continuation of drugs and diet used before trial
Medici *et al. *[57]	Stable chronic asthma	66 (0)	Unclear	PMOM: Peak expiratory flow variability baseline to 4 months	N:16 D:20 T:8w	Ac:C CA S:I C 1	Asthma drugs adapted if necessary^d^
Rampes *et al. *[60]	Addiction (alcohol abuse)	59 (54%)	Adequate^c^	PMOM: VAS craving for alcohol after 8 weeks	N: 6 D: 30 T: 6w	Ac:C EarA S:I C 2	Individual counseling and group therapy^d^
Röschke *et al. *[61]	Depression	70 (unclear)	Unclear	Responder according to Global Assessment Scale (GAS)	N: 12 D: 30 T: 4w	Ac:C CA S:I C 1	Mianserin 90 to 120 mg/day in all groups^d^
Smith *et al. *[63]	Nausea and vomiting during pregnancy	445 (25%)	Adequate	Rhodes Index of Nausea after 4 weeks	N: 5 D: 20 T: 4w	Ac:A CA S:I C 1	Pretrial treatment continued; lifestyle recommendations
Tremeau *et al. *[65]	Cervical maturation 37th to 38th pregnancy week	98 (18%)	Unclear	PMOM: Bishop score baseline to 10 days	N: 3 D: 20 T: 1w	Ac:C CA S:I C 1	None
Wang *et al. *[66]	Low-back and pelvic pain during pregnancy	159 (4%)	Adequate^c^	PMOM: VAS pain change after 1 week^c^	N: 1 D:^c^ T: 1w	Ac:C EA S:I C 2	Acetaminophen and other self-care if needed
Worner *et al. *[68]	Addiction (alcohol abuse)	56 (95%)	Unclear	Patients with either relapse or inpatient detoxification	N: n.i. D: 30 T: 12w	Ac:C CA S:II C 1	Counseling and group therapy^d^

^a^See Table 1 footnotes for definitions of abbreviations ^b^Two sham groups with different context. ^c^Additional information received from author. ^d^Classified as intense cointervention likely to influence the outcome.

n.i. = no information reported

Fifteen studies had a clearly predefined main outcome measure. For 32 trials, a continuous effect size measure could be calculated, and for 24 trials a dichotomous effect size measure (for 19 trials both a continuous and a dichotomous effect size measure could be calculated). Acupuncture interventions varied strongly regarding number of sessions, type of acupuncture (that is, classical acupuncture, electroacupuncture, ear acupuncture), level of individualization for point selection and number of needles used. In 31 trials, the sham procedure involved skin penetration (in 7 trials at acupuncture points not indicated for the condition treated and in 24 trials outside known acupuncture points); six trials used approaches without skin penetration (in three trials at the same points as in the acupuncture group and in three trials outside known points).

### Meta-analysis of nonspecific effects (sham acupuncture vs no acupuncture)

**Figure 2:**
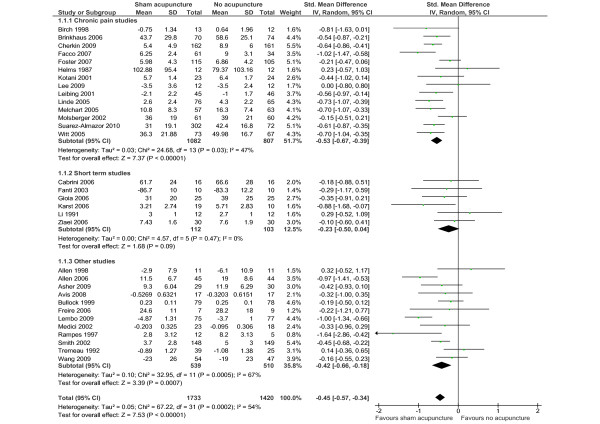
**The nonspecific effect of acupuncture (difference between groups receiving sham acupuncture and no acupuncture)**. SD, standard deviation; Total, number of patients; 95% CI, 95% confidence interval; IV, inverse variance method; Random, random effects model; df, degrees of freedom.

**Figure 3:**
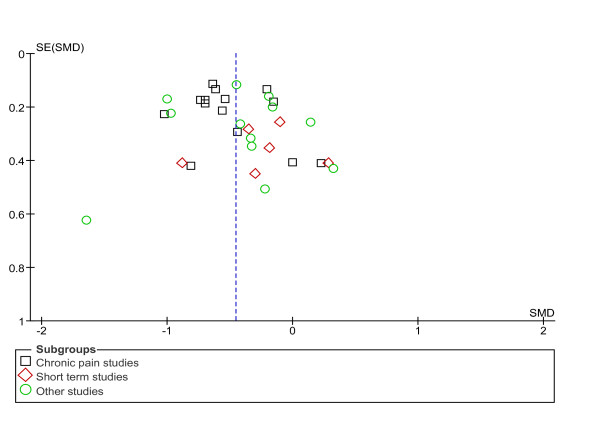
**Funnel plot of studies comparing sham acupuncture versus no acupuncture**. SE, standard error; SMD, standardized mean difference.

The main analyses are based on the 32 trials reporting data on a continuous outcome. For the comparison of sham acupuncture with no acupuncture, the pooled SMDs were -0.53 (95% CI -0.67, -0.39) among chronic pain trials, -0.23 (-0.50, 0.04) among short-term studies and -0.42 (95% CI -0.66, -0.18) in other studies (Figure 2). The test for differences between diagnostic subgroups missed statistical significance at the 5% level (*P *= 0.08). Effect sizes showed moderate statistical heterogeneity among chronic pain studies, no heterogeneity among short-term studies and marked heterogeneity among the other studies. If studies were pooled across clinical subgroups, the SMD was -0.45 (95% CI -0.57, -0.34). In seven trials, effects over no-treatment groups were large (SMDs were above -0.7); in nine trials, these effects were moderate (between -0.4 and -0.7); and in 16 trials, these effects were small (< -0.4). Results were similar when borderline studies were included, when in studies without a predefined main outcome measure other outcomes were chosen or when dichotomous outcomes were analyzed (see Additional file 1, Table S3). Egger's test did not suggest funnel plot asymmetry (*P *= 0.25; asymmetry coefficient 0.21) (Figure 3). In exploratory subgroup analyses (see Additional file 1, Table S3), effect sizes differed significantly according to the level of cointervention (larger if less cointerventions) and according to the type of sham intervention (larger if no skin penetration). Nonspecific effects tended to be larger in trials with a larger sample size, a lower risk of bias, and a clearly predefined outcome, but the differences were not statistically significant. In multivariate meta-regression analyses, only the association with level of cointerventions approached statistical significance (*P *= 0.07). Trials with larger effects of sham over no acupuncture reported smaller effects of acupuncture over sham intervention than trials with smaller nonspecific effects (β = -0.39, *P *= 0.029).

### Meta-analysis of specific effects (acupuncture vs sham acupuncture) and total effects (acupuncture vs no acupuncture)

For the comparison of acupuncture with sham acupuncture, the pooled random effects SMDs were -0.46 (95% CI -0.72, -0.20) for chronic pain studies, -0.34 (95% CI -0.79, 0.12) for short-term studies, and -0.28 (-0.59, 0.03) for other studies (see Additional file 1, Figure S1). There were no statistically significant (*P *= 0.71) differences between diagnostic subgroups, but there was substantial statistical heterogeneity in all three clinical categories. If trials were pooled across categories, the SMD was -0.37 (95% CI -0.55, -0.19). The funnel plot was highly asymmetrical (Additional file 1, Figure S2; *P *= 0.002; asymmetry coefficient -0.52). Larger trials yielded significantly less positive results than smaller trials (SMDs -0.15 (95% CI -0.31, 0.01) vs -0.59 (95% CI -0.93, -0.24); *P *< 0.001). Specific effects were also smaller in trials with lower risk of bias and more intense cointerventions, while skin penetration and condition did not have a significant influence.

The pooled SMDs between acupuncture and no acupuncture were -0.94 (95% CI -1.20, -0.67) for chronic pain studies, -0.60 (95% CI -1.08, -0.12) for short-term studies, and -0.63 (-0.91, -0.35) for other studies (see Additional file 1, Figure S3) with marked heterogeneity in all three categories. If all studies were pooled, the SMD was -0.77 (95% CI -0.94, -0.59). There was significant funnel plot asymmetry (*P *= 0.03; asymmetry coefficient -0.38), with smaller studies yielding larger effect estimates (Additional file 1, Figure S4, for the funnel plot).

## Discussion

### Summary of main findings

According to our findings (sham) acupuncture interventions are often associated with noteworthy nonspecific effects. Differences between sham acupuncture and no-acupuncture groups tended to be smaller in trials in which there were intense cointerventions in all study groups. Indicators of study quality (that is, sample size, risk of bias, predefinition of a main outcome measure) were not associated significantly with effect size. Trials with larger effects of sham over no acupuncture reported smaller effects of acupuncture over sham intervention than trials with smaller nonspecific effects. In our analyses, we also found small to moderate specific effects of acupuncture interventions over sham acupuncture; however, trials with large sample size and low risk of bias yielded less positive results. In our study set, the total effect of acupuncture interventions including both specific and nonspecific effects was, on average, at least moderate in size.

### Strengths and limitations

Although we did not systematically search Chinese language databases, our review is currently the most comprehensive and largest analysis of randomized trials of acupuncture including both a sham and a no-treatment control group. It includes many more and larger trials than previous analyses [26-28]. The overall findings are highly robust to sensitivity analyses and indicators of study quality. The most important limitation of our review is the strong heterogeneity of our trial set regarding patients, interventions, outcomes and methodological quality. We do not think that pooling such a heterogeneous set of studies would be adequate if the aim were primarily to assess effectiveness for clinical decision making. However, our primary aim was to investigate whether (sham) acupuncture interventions are, on average, associated with relevant nonspecific effects. To assess the size of nonspecific effects, it is necessary to include trials with both a sham and a no-acupuncture control group. As the number of such trials is limited, pooling all available information can be justified for generating hypotheses and has been performed in the Cochrane review on placebo effects in all conditions in a much more radical manner [26].

The comparisons between sham acupuncture and acupuncture in the primary studies included in our review are unblinded. As almost all trials focused on patient-reported outcome measures, there is considerable risk of bias. Patients randomized to the no-treatment group might be disappointed and experience "nocebo" effects, or they might give overly negative ratings for subjective symptoms. On the other hand, patients randomized to no-treatment groups might use larger doses of rescue medication or cointerventions which would lead to an underestimation of the differences. In fact, in some of the trials included in our review, patients in no-acupuncture control groups had higher analgesic use than patients in the sham groups (for example, [56,58]). Insufficient blinding is also a problem for the comparison between acupuncture and placebo acupuncture [28]. However, if patients find out that they are in a sham group, one would expect an underestimation of the effect of sham over no treatment. In summary, it is difficult to assess to what extent and in which direction biases can distort effect estimates between sham and no-acupuncture groups. It is noteworthy that although indicators of study quality were not significantly associated with the size of nonspecific effects, better and larger studies tended to report larger effects. It seems that our estimate of nonspecific effects is less subject to small study bias and other biases than those for specific and total effects.

### Interpretation

Our findings are highly consistent with smaller analyses available in the literature [27,28]. The reanalysis of the 21 acupuncture trials included in the Cochrane review on placebo effects yielded a SMD of -0.41 [27]. Owing to slightly different inclusion criteria, five trials were excluded from the current analyses. A meta-analysis by Madsen *et al. *[28], who reviewed 13 three-armed trials on acupuncture for acute and chronic pain, found a SMD of -0.42. Nine of the studies included in their review were also included in our review, while we excluded four trials due to slightly different selection criteria. Our main analysis includes 23 additional trials (including seven trials addressing chronic or acute pain).

It has been argued that sham interventions in which needles penetrate the skin (particularly if applied in the same dermatomes as the true acupuncture intervention) are physiologically not inert and therefore should not be considered as placebos [10]. Our exploratory subgroup analyses (as well as similar analyses in the review by Madsen *et al. *[28]) do not provide evidence that sham interventions involving needle penetration are associated with larger nonspecific effects than those which do not. Thus the limited available data suggest that skin penetration or no skin penetration does not seem to make a big difference.

If acupuncture should have indeed relevant total effects but only very limited specific effects, this would have major implications for the conduct and interpretation of clinical trials. On the basis of our data and available systematic reviews [4-7,28], it seems reasonable to assume an average SMD of 0.4 (or more) for nonspecific effects and SMD of 0.2 (or less) for specific effects at least for a number of conditions. To achieve 80% power, a two-armed, sham-controlled clinical trial investigating a specific effect of 0.2 SMD would have to recruit about 800 patients. This suggests that almost all available trials comparing true and sham acupuncture would be underpowered.

One could argue that a SMD of 0.2 is clinically irrelevant. In line with that reasoning, Madsen *et al. *[28] questioned in their review whether "the prevailing hypothesis that acupuncture has an important effect on pain in general." (page 7). However, we believe that another conclusion is possible, too. As we did, Madsen *et al. *found, on average, a moderately large effect of sham interventions over no-acupuncture groups, and both reviews found at least small specific effects of acupuncture over sham interventions. The total effect of acupuncture seems to be at least moderate in size in a number of conditions, and such effects can well be clinically relevant. For many established drug treatments, SMDs over placebo are in the range between 0.3 and 0.5 (for example, [77,78]). If, as the available data suggest [26], clinical effects associated with pharmacological placebos are small compared to no treatment (with a SMD of 0.1 on average), the total effects of these treatments could be in a similar range (around a SMD of 0.4 to 0.6) as those of several acupuncture interventions. It could be argued that for a suffering individual, it does not matter whether relief is due to specific or nonspecific effects. However, as the evidence for larger nonspecific effects of acupuncture compared to other treatments comes with one exception [23] from indirect comparisons open to confounding, firm conclusions are not yet possible.

We think that our findings are of major relevance to the question how the clinical effectiveness of complex nondrug interventions should be assessed. It is likely that nonspecific effects vary between different types of complex treatment interventions. The concept of specific and nonspecific effects might not be fully adequate in that case, as so-called nonspecific effects might turn out to be characteristic for a given therapeutic setting. If the total effect of an intervention in clinical practice would indeed consist of variable contributions of specific and nonspecific effects, it could be that a treatment which has only minor or even no specific but clinically relevant nonspecific effects has a larger total effect than a treatment with moderate specific but only minor nonspecific effects. This has been denoted the efficacy paradox [79]. Should such a treatment be readily available? The position of a pragmatic decision maker could be yes if the comparative treatment represents adequate standard treatment. In fact, in Germany, acupuncture is routinely reimbursed for chronic low-back pain as in a large randomized trial acupuncture (but also sham acupuncture which is not reimbursed) was more effective than treatment based on German guidelines [3]. Skeptical scientists would argue that these results are likely to be biased because of lack of blinding and that acupuncture should not be considered effective. Furthermore, if issues such as expectancies, beliefs and trust should have a relevant influence on the effectiveness of a treatment, the findings of clinical trials might no longer be valid when attitudes in a population change over time.

## Conclusions

Sham acupuncture interventions are often associated with moderately large nonspecific effects, which could make it difficult to detect small additional specific effects. Compared to inert placebo interventions, effects associated with sham acupuncture might be larger, which would have considerable implications for the design and interpretation of clinical trials. Total effects of acupuncture interventions including both specific and nonspecific effects often seem to be at least moderate in size. We believe that there has to be a discussion involving scientists, decision makers, health care providers and patients whether and when the evidence for clinically relevant total effects from nonblinded comparisons is sufficient to consider a treatment effective, even if specific effects due to the postulated mechanism of action might be minor or even nonexistent.

## Competing interests

KL received travel reimbursement and fees for speaking at conferences organized by acupuncture societies in the USA, UK, Germany, Japan and Spain. Antonius Schneider received fees for lecturing for a German acupuncture society (DÄGfA) until 2006. KM and KN do not have any conflicts of interest.

## Authors' contributions

KL, KN and KM were involved in the literature search, data extraction and analysis. AS provided advice on acupuncture and participated in the interpretation of the data. KL conceived and coordinated the study and wrote the first draft of the manuscript. All authors commented on drafts and approved the final manuscript.

## Pre-publication history

The pre-publication history for this paper can be accessed here:

http://www.biomedcentral.com/1741-7015/8/75/prepub

## Supplementary Material

Additional file 1 **Search strategy MEDLINE 19 April 2010 (1966-2010, week 15)**. Search strategy. Embase search, 19 April 2010 (1988-2010, week 15). Table S1. Additional publications related to included studies. Table S2. Excluded studies. Table S3. Subgroup and sensitivity analyses. Figure S1. The "specific" effect of acupuncture (difference between groups receiving acupuncture and sham acupuncture). Figure S2. Funnel plot of studies comparing acupuncture versus sham acupuncture. Figure S3. The "total" effect of acupuncture (difference between groups receiving acupuncture and no acupuncture). Figure S4. Funnel plot of studies comparing acupuncture versus no acupuncture. Click here for file

## References

[B1] Jena S Witt CM Brinkhaus B Wegscheider K Willich SN Acupuncture in patients with headache Cephalalgia 2008 28 969 979 10.1111/j.1468-2982.2008.01640.x 18624803

[B2] Witt CM Jena S Selim D Brinkhaus B Reinhold T Wruck K Liecker B Linde K Wegscheider K Willich SN Pragmatic randomized trial evaluating the clinical and economic effectiveness of acupuncture for chronic low back pain Am J Epidemiol 2006 164 487 496 10.1093/aje/kwj224 16798792

[B3] Haake M Muller HH Schade-Brittinger C Basler HD Schafer H Maier C Endres HG Trampisch HJ Molsberger A German Acupuncture Trials (GERAC) for chronic low back pain: randomized, multicenter, blinded, parallel-group trial with 3 groups Arch Intern Med 2007 167 1892 1898 10.1001/archinte.167.17.1892 17893311

[B4] Linde K Allais G Brinkhaus B Manheimer E Vickers A White AR Acupuncture for migraine prophylaxis Cochrane Database Syst Rev 2009 1 CD001218 19160193 10.1002/14651858.CD001218.pub2 PMC3099267

[B5] Linde K Allais G Brinkhaus B Manheimer E Vickers A White AR Acupuncture for tension-type headache Cochrane Database Syst Rev 2009 1 CD007587 19160338 10.1002/14651858.CD007587 PMC3099266

[B6] Manheimer E Linde K Lao L Bouter LM Berman BM Meta-analysis: acupuncture for osteoarthritis of the knee Ann Intern Med 2007 146 868 877 17577006 10.7326/0003-4819-146-12-200706190-00008

[B7] Yuan J Purepong N Kerr DP Park J Bradbury I McDonough S Effectiveness of acupuncture for low back pain: a systematic review Spine (Phila Pa 1976) 2008 33 E887 E900 18978583 10.1097/BRS.0b013e318186b276

[B8] Bausell RB Snake oil science: The truth about complementary and alternative medicine 2007 Oxford, UK: Oxford University Press

[B9] Birch S A review and analysis of placebo treatments, placebo effects, and placebo controls in trials of medical procedures when sham is not inert J Alternat Complement Med 2006 12 303 310 10.1089/acm.2006.12.303 16646730

[B10] Lund I Lundeberg T Are minimal, superficial or sham acupuncture procedures acceptable as inert placebo controls? Acupunct Med 2006 24 13 15 10.1136/aim.24.1.13 16618044

[B11] Kaptchuk TJ The placebo effect in alternative medicine: can the performance of a healing ritual have clinical significance? Ann Intern Med 2002 136 817 825 12044130 10.7326/0003-4819-136-11-200206040-00011

[B12] Liu T Yu CP Placebo analgesia, acupuncture and sham surgery eCAM 2010 in press 10.1093/ecam/neq030 PMC3139509 21785643

[B13] Shapiro AK Morris LA Garfield SL, Bergin AE The placebo effect in medical and psychological therapies Handbook of psychotherapy and behavior change 1978 2 New York: Wiley 369 410

[B14] Grünbaum A The placebo concept in medicine and psychiatry Psycholog Med 1986 16 19 38 10.1017/S0033291700002506 3515378

[B15] Finniss DG Kaptchuk TJ Miller F Benedetti F Biological, clinical, and ethical advances of placebo effects Lancet 2010 375 686 695 10.1016/S0140-6736(09)61706-2 20171404 PMC2832199

[B16] Ernst E Resch KL Concept of true and perceived placebo effects BMJ 1995 311 551 553 7663213 10.1136/bmj.311.7004.551 PMC2550609

[B17] Kienle GS Kiene H The powerful placebo effect: fact or fiction? J Clin Epidemiol 1997 50 1311 1318 10.1016/S0895-4356(97)00203-5 9449934

[B18] Vickers AJ de Craen AJM Why use placebos in clinical trials? A narrative review of the methological literature J Clin Epidemiol 2000 53 157 161 10.1016/S0895-4356(99)00139-0 10729687

[B19] Wampold BE Minami T Tierney SC Baskin TW Bhati KS The placebo is powerful: estimating placebo effects in medicine and psychotherapy from randomized clinical trials J Clin Psychol 2005 61 835 854 10.1002/jclp.20129 15827993

[B20] Napadow V Ahn A Longhurst J Lao L Stener-Victorin E Harris R Langevin HM The status and future of acupuncture mechanism research J Altern Complement Med 2008 14 861 869 10.1089/acm.2008.SAR-3 18803495 PMC3155097

[B21] Dincer F Linde K Sham interventions in randomized clinical trials of acupuncture: a review Complement Ther Med 2003 11 235 242 10.1016/S0965-2299(03)00124-9 15022656

[B22] Kaptchuk TJ Kelley JM Conboy LA Davis RB Kerr CE Jacobson EE Kirsch I Schyner RN Nam BH Nguyen LT Park M Rivers AL McManus C Kokkotou E Drossman DA Goldman P Lembo AJ Components of placebo effect: randomised controlled trial in patients with irritable bowel syndrome BMJ 2008 336 999 1003 10.1136/bmj.39524.439618.25 18390493 PMC2364862

[B23] Kaptchuk TJ Stason WB Davis RB Legedza ATR Schnyer RN Kerr CE Stone DA Huyn Nam B Kirsch I Goldman RH Sham device vs. inert pill: randomised controlled trial of two placebo treatments BMJ 2006 332 391 397 10.1136/bmj.38726.603310.55 16452103 PMC1370970

[B24] Hrobjartsson A Gøtzsche PC Is the placebo powerless? an analysis of clinical trials comparing placebo with no treatment N Engl J Med 2001 344 1594 1602 10.1056/NEJM200105243442106 11372012

[B25] Hrobjartsson A Gøtzsche PC Placebo interventions for all clinical conditions Cochrane Database Syst Rev 2004 3 CD003974 15266510 10.1002/14651858.CD003974.pub2

[B26] Hrobjartsson A Gøtzsche PC Placebo interventions for all clinical conditions Cochrane Database Syst Rev 2010 1 CD003974 20091554 10.1002/14651858.CD003974.pub3 PMC7156905

[B27] Linde K Niemann K Meissner K Are sham acupuncture interventions more effective than (other) placebos? A re-analysis of data from the Cochrane review on placebo effects Forsch Komplementrmed 2010 17 259 264 10.1159/000320374 20980765

[B28] Madsen MV Gøtzsche PC Hrobjartsson A Acupuncture treatment for pain: systematic review of randomised clinical trials with acupuncture, placebo acupuncture, and no acupuncture groups BMJ 2009 338 a3115 10.1136/bmj.a3115 19174438 PMC2769056

[B29] Schünemann HJ Oxman AD Vist GE Higgins JPT Deeks JJ Glasziou P Guyatt GH Higgins JPT, Green S Interpreting results and drawing conclusions Cochrane Handbook for Systematic Reviews of Interventions 2008 The Cochrane Collaboration http://www.cochrane-handbook.org/

[B30] Deeks JJ Altman DG Bradburn MJ Egger M, Smith GD, Altman DG Statistical methods for examining heterogeneity and combining results from several studies in meta-analysis Sytematic reviews in health care: Meta-analysis in context 2001 London: BMJ Books 285 312

[B31] Egger M Davey SG Schneider M Minder C Bias in meta-analysis detected by a simple, graphical test BMJ 1997 315 629 634 9310563 10.1136/bmj.315.7109.629 PMC2127453

[B32] Wilson DB SPSS, Stata, and SAS macros for performing meta-analytic analyses 2010 http://mason.gmu.edu/~dwilsonb/ma.html

[B33] Allen JJB Schnyer R Hitt SK The efficacy of acupuncture in the treatment of major depression in women Psychol Sci 1998 9 397 401 10.1111/1467-9280.00074

[B34] Allen JJB Schnyer RN Chambers AS Hitt SK Moreno FA Manber R Acupuncture for depression: a randomized controlled trial J Clin Psychiatry 2006 67 1665 1673 10.4088/JCP.v67n1101 17196044

[B35] Asher GN Coeytaux RR Chen W Reilly AC Loh YL Harper TC Acupuncture to initiate labor (Acumoms 2): a randomized, sham-controlled clinical trial J Matern Fetal Neonatal Med 2009 22 843 848 10.1080/14767050902906386 19526433 PMC2919333

[B36] Aune A Alraek T LiHua H Baerheim A Acupuncture in the prophylaxis of recurrent lower urinary tract infection in adult women Scand J Prim Health Care 1998 16 37 39 10.1080/028134398750003386 9612877

[B37] Avis NE Legault C Coeytaux RR Pian-Smith M Shifren JL Chen W Valaskatgis P A randomized, controlled pilot study of acupuncture treatment for menopausal hot flashes Menopause 2008 15 1070 1078 10.1097/gme.0b013e31816d5b03 18528313

[B38] Birch S Jamison RN Controlled trial of Japanese acupuncture for chronic myofascial neck pain: assessment of specific and nonspecific effects of treatment Clin J Pain 1998 14 248 255 10.1097/00002508-199809000-00012 9758075

[B39] Brinkhaus B Witt CM Jena S Linde K Streng A Wagenpfeil S Irnich D Walther HU Melchart D Willich SN Acupuncture in patients with chronic low back pain: a randomized controlled trial Arch Intern Med 2006 166 450 457 10.1001/.450 16505266

[B40] Bullock ML Kiresuk TJ Pheley AM Culliton PD Lenz SK Auricular acupuncture in the treatment of cocaine abuse: a study of efficacy and dosing J Subst Abuse Treat 1999 16 31 38 10.1016/S0740-5472(98)00002-6 9888119

[B41] Cabrini L Gioia L Gemma M Melloni G Carretta A Ciriaco P Puglisi A Acupuncture for diagnostic fiberoptic bronchoscopy: a prospective, randomized, placebo-controlled study Am J Chin Med 2006 34 409 415 10.1142/S0192415X06003941 16710890

[B42] Cherkin DC Sherman KJ Avins AL Erro JH Ichikawa L Barlow WE Delaney K Hawkes R Hamilton L Pressman A Khalsa PS Deyo RA A randomized trial comparing acupuncture, simulated acupuncture, and usual care for chronic low back pain Arch Intern Med 2009 169 858 866 10.1001/archinternmed.2009.65 19433697 PMC2832641

[B43] Dundee JW Chestnutt WN Ghaly RG Lynas AG Traditional Chinese acupuncture: a potentially useful antiemetic? Br Med J (Clin Res Ed) 1986 293 583 584 10.1136/bmj.293.6547.583 3092933 PMC1341376

[B44] Facco E Liguori A Petti F Zanette G Coluzzi F De Nardin M Mattia C Traditional acupuncture in migraine: a controlled, randomized study Headache 2008 48 398 407 10.1111/j.1526-4610.2007.00916.x 17868354

[B45] Fanti L Gemma M Passaretti S Guslandi M Testoni PA Casati A Torri G Electroacupuncture analgesia for colonoscopy. a prospective, randomized, placebo-controlled study Am J Gastroenterol 2003 98 312 316 12591047 10.1111/j.1572-0241.2003.07231.x

[B46] Foster NE Thomas E Barlas P Hill JC Young J Mason E Hay EM Acupuncture as an adjunct to exercise based physiotherapy for osteoarthritis of the knee: randomised controlled trial BMJ 2007 335 436 10.1136/bmj.39280.509803.BE 17699546 PMC1962890

[B47] Freire AO Sugai GCM Chrispin FS Togeiro SM Yamamura Y Mello LE Tufik S Treatment of moderate obstructive sleep apnea syndrome with acupuncture: a randomised, placebo-controlled pilot trial Sleep Med 2007 8 43 50 10.1016/j.sleep.2006.04.009 17023212

[B48] Gioia L Cabrini L Gemma M Fiori R Fasce F Bolognesi G Spinelli A Beretta L Sedative effect of acupuncture during cataract surgery: prospective randomized double-blind study J Cataract Refract Surg 2006 32 1951 1954 10.1016/j.jcrs.2006.06.027 17081902

[B49] Helms JM Acupuncture for the management of primary dysmenorrhea Obstet Gynecol 1987 69 51 56 3540764

[B50] Karst M Winterhalter M Munte S Francki B Hondronikos A Eckardt A Hoy L Buhck H Bernateck M Fink M Auricular acupuncture for dental anxiety: a randomized controlled trial Anesth Analg 2007 104 295 300 10.1213/01.ane.0000242531.12722.fd 17242083

[B51] Kotani N Kushikata T Suzuki A Hashimoto H Muraoka M Matsuki A Insertion of intradermal needles into painful points provides analgesia for intractable abdominal scar pain Reg Anesth Pain Med 2001 26 532 538 11707792 10.1053/rapm.2001.25897

[B52] Lee SH Lee BC Electroacupuncture relieves pain in men with chronic prostatitis/chronic pelvic pain syndrome: three-arm randomized trial Urology 2009 73 1036 1041 10.1016/j.urology.2008.10.047 19394499

[B53] Leibing E Leonhardt U Koster G Goerlitz A Rosenfeldt JA Hilgers R Ramadori G Acupuncture treatment of chronic low-back pain: a randomized, blinded, placebo-controlled trial with 9-month follow-up Pain 2002 96 189 196 10.1016/S0304-3959(01)00444-4 11932074

[B54] Lembo AJ Conboy L Kelley JM Schnyer RS McManus CA Quilty MT Kerr CE Drossman D Jacobson EE Davis RB A treatment trial of acupuncture in IBS patients Am J Gastroenterol 2009 104 1489 1497 10.1038/ajg.2009.156 19455132 PMC2694961

[B55] Li CK Nauck M Loser C Folsch UR Creutzfeldt W [Acupuncture to alleviate pain during colonoscopy] Dtsch Med Wochenschr 1991 116 367 370 10.1055/s-2008-1063621 2001639

[B56] Linde K Streng A Jurgens S Hoppe A Brinkhaus B Witt C Wagenpfeil S Pfaffenrath V Hammes MG Weidenhammer W Willich SN Melchart D Acupuncture for patients with migraine: a randomized controlled trial JAMA 2005 293 2118 2125 10.1001/jama.293.17.2118 15870415

[B57] Medici TC Grebski E Wu J Hinz G Wuthrich B Acupuncture and bronchial asthma: a long-term randomized study of the effects of real versus sham acupuncture compared to controls in patients with bronchial asthma J Altern Complement Med 2002 8 737 750 10.1089/10755530260511748 12614526

[B58] Melchart D Streng A Hoppe A Brinkhaus B Witt C Wagenpfeil S Pfaffenrath V Hammes M Hummelsberger J Irnich D Weidenhammer W Willich SN Linde K Acupuncture in patients with tension-type headache: randomised controlled trial BMJ 2005 331 376 382 10.1136/bmj.38512.405440.8F 16055451 PMC1184247

[B59] Molsberger AF Mau J Pawelec DB Winkler J Does acupuncture improve the orthopedic management of chronic low back pain: a randomized, blinded, controlled trial with 3 months follow up Pain 2002 99 579 587 10.1016/S0304-3959(02)00269-5 12406534

[B60] Rampes H Pereira S Mortimer A Manoharan S Knowles M Does electroacupuncture reduce craving for alcohol? a randomized controlled study Complement Ther Med 1997 5 19 26 10.1016/S0965-2299(97)80085-4

[B61] Röschke J Wolf C Muller MJ Wagner P Mann K Grozinger M Bech S The benefit from whole body acupuncture in major depression J Affect Disord 2000 57 73 81 10.1016/S0165-0327(99)00061-0 10708818

[B62] Rusy LM Hoffman GM Weisman SJ Electroacupuncture prophylaxis of postoperative nausea and vomiting following pediatric tonsillectomy with or without adenoidectomy Anesthesiology 2002 96 300 305 10.1097/00000542-200202000-00013 11818760

[B63] Smith C Crowther C Beilby J Acupuncture to treat nausea and vomiting in early pregnancy: a randomized controlled trial Birth 2002 29 1 9 10.1046/j.1523-536X.2002.00149.x 11843784

[B64] Suarez-Almazor ME Looney C Liu Y Cox V Pietz K Donald M Street R A randomized controlled trial of acupuncture for osteoarthritis of the knee: effects of patient-provider communication Arthritis Care Res 2010 62 1229 1236 10.1002/acr.20225 PMC3651275 20506122

[B65] Tremeau ML Fontanie-Ravier P Teurnier F Demouzon J [Protocol of cervical maturation by acupuncture] J Gynecol Obstet Biol Reprod (Paris) 1992 21 375 380 1624722

[B66] Wang SM Dezinno P Lin EC Lin H Yue JJ Berman MR Braveman F Kain ZN Auricular acupuncture as a treatment for pregnant women who have low back and posterior pelvic pain: a pilot study Am J Obstet Gynecol 2009 201 271 e271-279 19560110 10.1016/j.ajog.2009.04.028 PMC2768290

[B67] Witt C Brinkhaus B Jena S Linde K Streng A Wagenpfeil S Hummelsberger J Walther HU Melchart D Willich SN Acupuncture in patients with osteoarthritis of the knee: a randomised trial Lancet 2005 366 136 143 10.1016/S0140-6736(05)66871-7 16005336

[B68] Worner TM Zeller B Schwarz H Zwas F Lyon D Acupuncture fails to improve treatment outcome in alcoholics Drug Alcohol Depend 1992 30 169 173 10.1016/0376-8716(92)90022-5 1633756

[B69] Ziaei S Hayipour L Effect of acupuncture on labor Intern J Gynecol Obstet 2006 92 71 72 10.1016/j.ijgo.2005.09.008 16253255

[B70] Benson MR Elkind-Hirsch KE Theall A Fong K Hogan RB Scott RT Impact of acupuncture before and after embryo transfer on the outcome of in vitro fertilization cycles: a prospective single-blind randomized study Fertil Steril 2006 86 Suppl 1 S135 10.1016/j.fertnstert.2006.07.362

[B71] Fratterelli JL Leondires MR Fong K Theall A Locatelli S Scott RT Laser acupuncture before and after embryo transfer imporves ART delivery rates: results of a prospective randomized double-blinded placebo controlled five-armed trial involving 1000 patients Fertil Steril 2008 90 Suppl 1 S105 10.1016/j.fertnstert.2008.07.1252

[B72] Avants SK Margolin A Holford TR Kosten TR A randomized controlled trial of auricular acupuncture for cocaine dependence Arch Intern Med 2000 160 2305 2312 10.1001/archinte.160.15.2305 10927727

[B73] Berman BM Lao L Langenberg P Lee WL Gilpin AMK Hochberg MC Effectiveness of acupuncture as adjunctive therapy in osteoarthritis of the knee: a randomized, controlled trial Ann Intern Med 2004 141 901 910 15611487 10.7326/0003-4819-141-12-200412210-00006

[B74] Margolin A Kleber HD Avants SK Konefal J Gawin F Stark E Sorensen J Midkiff E Wells E Jackson TR Bullock M Culliton PD Boles S Vaughan R Acupuncture for the treatment of cocaine addiction: a randomized controlled trial JAMA 2002 287 55 63 10.1001/jama.287.1.55 11754709

[B75] Scharf HP Mansmann U Streitberger K Witte S Kramer J Maier C Trampisch HJ Victor N Acupuncture and knee osteoarthritis: a three-armed randomized trial Ann Intern Med 2006 145 12 20 16818924 10.7326/0003-4819-145-1-200607040-00005

[B76] Shen J Wenger N Glaspy J Hays RD Albert PS Choi C Shekelle PG Electroacupuncture for control of myeloablative chemotherapy-induced emesis: a randomized controlled trial JAMA 2000 284 2755 2761 10.1001/jama.284.21.2755 11105182

[B77] Bjordal JM Ljunggren AE Klovning A Slordal L Non-steroidal anti-inflammatory drugs, including cyclo-oxygenase-2 inhibitors, in osteoarthritic knee pain: meta-analysis of randomised placebo controlled trials BMJ 2004 329 1317 10.1136/bmj.38273.626655.63 15561731 PMC534841

[B78] Turner EH Matthews AM Linardatos E Tell RA Rosenthal R Selective publication of antidepressant trials and its influence on apparent efficacy N Engl J Med 2008 358 252 260 10.1056/NEJMsa065779 18199864

[B79] Walach H The efficacy paradox in randomized controlled trials of CAM and elsewhere: beware of the placebo trap J Altern Complement Med 2001 7 213 218 10.1089/107555301300328070 11439833

[B80] Aune A Alraek T Huo L Baerheim A [Can acupuncture prevent cystitis in women?] Tidsskr Nor Laegeforen 1998 118 9 1370 1372 9599500

[B81] Brinkhaus B Becker-Witt C Jena S Linde K Streng A Wagenpfeil S Irnich D Hummelsberger J Melchart D Willich SN Acupuncture Randomized Trials (ART) in patients with chronic low back pain and osteoarthritis of the knee: design and protocols Forsch Komplementarmed Klass Naturheilkd 2003 10 185 191 10.1159/000073474 12972723

[B82] Brinkhaus B Witt CM Jena S Linde K Streng A Irnich D Hummelsberger J Hammes M Pach D Melchart D Willich SN Interventions and physician characteristics in a randomized multicenter trial of acupuncture in patients with low-back pain J Altern Complement Med 2006 12 649 657 10.1089/acm.2006.12.649 16970535

[B83] Brinkhaus B Witt CM Jena S Linde K Streng A Hummelsberger J Irnich D Hammes M Pach D Melchart D Willich SN Physician and treatment characteristics in a randomised multicentre trial of acupuncture in patients with osteoarthritis of the knee Complement Ther Med 2007 15 180 189 10.1016/j.ctim.2006.04.003 17709063

[B84] Hay E Barlas P Foster N Hill J Thomas E Young J Is acupuncture a useful adjunct to physiotherapy for older adults with knee pain?: the "acupuncture, physiotherapy and exercise" (APEX) study [ISRCTN88597683] BMC Musculoskelet Disord 2004 5 31 10.1186/1471-2474-5-31 15345098 PMC520743

[B85] Linde K Streng A Hoppe A Brinkhaus B Witt CM Hammes M Irnich D Hummelsberger J Willich SN Melchart D Treatment in a randomized multicenter trial of acupuncture for migraine (ART migraine) Forsch Komplementarmed 2006 13 101 108 10.1159/000091999 16645290

[B86] Melchart D Linde K Streng A Reitmayr S Hoppe A Brinkhaus B Becker-Witt C Wagenpfeil S Pfaffenrath V Hammes M Willich SN Weidenhammer W Acupuncture randomized trials (ART) in patients with migraine or tension-type headache: design and protocols Forsch Komplementarmed Klass Naturheilkd 2003 10 179 184 10.1159/000073473 12972722

[B87] Melchart D Streng A Hoppe A Linde K Brinkhaus B Becker-Witt C Willich SN Hammes M Irnich D Hummelsberger J The acupuncture randomised trial (ART) for tension-type headache: details of the treatment Acupunct Med 2005 23 157 165 10.1136/aim.23.4.157 16430123

[B88] Roschke J Wolf C Kogel P Wagner P Bech S [Adjuvant whole body acupuncture in depression: a placebo-controlled study with standardized mianserin therapy] Nervenarzt 1998 69 961 967 10.1007/s001150050370 9859117

[B89] Smith C Crowther C The placebo response and effect of time in a trial of acupuncture to treat nausea and vomiting in early pregnancy Complement Ther Med 2002 10 210 216 10.1016/S0965-2299(02)00072-9 12594971

[B90] Smith C Crowther C Beilby J Pregnancy outcome following women's participation in a randomised controlled trial of acupuncture to treat nausea and vomiting in early pregnancy Complement Ther Med 2002 10 78 83 10.1054/ctim.2002.0523 12481955

[B91] Bier ID Wilson J Studt P Shakleton M Auricular acupuncture, education, and smoking cessation: a randomized, sham-controlled trial Am J Public Health 2002 92 1642 1647 10.2105/AJPH.92.10.1642 12356614 PMC1447300

[B92] Bullock ML Kiresuk TJ Sherman RE Lenz SK Culliton PD Boucher TA Nolan CJ A large randomized placebo controlled study of auricular acupuncture for alcohol dependence J Subst Abuse Treat 2002 22 71 77 10.1016/S0740-5472(01)00217-3 11932132

[B93] Chow OKW So SY Lam WK Effect of acupuncture on exercise-induced asthma Lung 1983 161 321 326 10.1007/BF02713881 6645620

[B94] Cottraux J Schbath J Messy P Mollard E Juenet C Collet L Predictive value of MMPI scales on smoking cessation programs outcomes Acta Psychiatr Belg 1986 86 463 469 3788644

[B95] Cottraux JA Harf R Boissel JP Smoking cessation with behaviour therapy or acupuncture: a controlled study Behav Res Ther 1983 21 417 424 10.1016/0005-7967(83)90011-6 6626112

[B96] Dundee JW Ghaly RG Bill KM Chestnutt WN Fitzpatrick KT Lynas AG Effect of stimulation of the P6 antiemetic point on postoperative nausea and vomiting Br J Anaesth 1989 63 612 618 10.1093/bja/63.5.612 2605083

[B97] Fung KP Chow OK So SY Attenuation of exercise-induced asthma by acupuncture Lancet 1986 2 1419 1422 2878275

[B98] Gerardi AU Dominici S Sapia F Reflexotherapy in respiratory allergies [in Italian] Minerva Med 1983 74 2521 2531 6361613

[B99] Gosman-Hedstrom G Claesson L Klingenstierna U Carlsson J Olausson B Frizell M Fagerberg B Blomstrand C Effects of acupuncture treatment on daily life activities and quality of life: a controlled, prospective, and randomized study of acute stroke patients Stroke 1998 29 2100 2108 9756589 10.1161/01.str.29.10.2100

[B100] Lin JG Lo MW Wen YR Hsieh CL Tsai SK Sun WZ The effect of high and low frequency electroacupuncture in pain after lower abdominal surgery Pain 2002 99 509 514 10.1016/S0304-3959(02)00261-0 12406527

[B101] Lin ZP Lan LW He TY Lin SP Lin JG Jang TR Ho TJ Effects of acupuncture stimulation on recovery ability of male elite basketball athletes Am J Chin Med 2009 37 471 481 10.1142/S0192415X09006989 19606508

[B102] Ludwig M Acupuncture in rehabilitative strength training: Spontaneous improvement of strength and EMG values of the quadricep muscles after anterior cruciate ligament reconstruction [in German] Deutsche Zeitschrift fur Akupunktur 1999 42 144 148

[B103] Rosler A Otto B Schreiber-Dietrich D Steinmetz H Kessler KR Single-needle acupuncture alleviates gag reflex during transesophageal echocardiography: a blinded, randomized, controlled pilot trial J Altern Complement Med 2003 9 847 849 10.1089/107555303771952190 14736356

[B104] Sertel S Herrmann S Greten HJ Haxsen V El-Bitar S Simon CH Baumann I Plinkert PK Additional use of acupuncture to NSAID effectively reduces post-tonsillectomy pain Eur Arch Otorhinolaryngol 2009 266 919 925 10.1007/s00405-008-0851-1 18982338

[B105] Sprott HM Mennet p Stratz T Müller P Wirksamkeit der Akupunktur bei Patienten mit generalisierter Tendomyopathie (Fibromyalgie) Akt Rheumatol 1993 18 132 135 10.1055/s-2008-1047326

[B106] Sprott H Efficiency of acupuncture in patients with fibromyalgia Clin Bull Myofascial Ther 1998 3 37 43 10.1300/J425v03n01_05

[B107] Tashkin DP Bresler DE Kroening RJ Kerschner H Katz RL Coulson A Comparison of real and simulated acupuncture and isoproterenol in methacholine-induced asthma Ann Allergy 1977 39 379 387 339785

[B108] Kim JI Lee MS Jung SY Choi JY Lee S Ko JM Zhao H Zhao J Kim AR Shin MS Kang KW Jung HJ Kim TH Liu B Choi SM Acupuncture for persistent allergic rhinitis: a multi-centre, randomised, controlled trial protocol Trials 2009 10 54 10.1186/1745-6215-10-54 19602250 PMC2715403

[B109] Vas J Rebollo A Perea-Milla E Méndez C Font CR Gómez-Río M Martín-Avila M Carbrera-Iboleón J Caballero MD Olmos MA Aguilar I Faus V Martos F Study protocol for a pragmatic randomised controlled trial in general practice investigating the effectiveness of acupuncture against migraine BMC Complement Altern Med 2008 8 12 10.1186/1472-6882-8-12 18410686 PMC2377233

