# Supplementary material for: How large are the nonspecific effects of acupuncture? A meta-analysis of randomized controlled trials
Source: BMC Med. 2010 Nov 23;8:75. doi: 10.1186/1741-7015-8-75 (PMC3001416; doi:10.1186/1741-7015-8-75)
Supplement: Additional file 1 — Search strategy MEDLINE 19 April 2010 (1966-2010, week 15). Search strategy. Embase search, 19 April 2010 (1988-2010, week 15). Table S1. Additional publications related to included studies. Table S2. Excluded studies. Table S3. Subgroup and sensitivity analyses. Figure S1. The "specific" effect of acupuncture (difference between groups receiving acupuncture and sham acupuncture). Figure S2. Funnel plot of studies comparing acupuncture versus sham acupuncture. Figure S3. The "total" effect of acupuncture (difference between groups receiving acupuncture and no acupuncture). Figure S4. Funnel plot of studies comparing acupuncture versus no acupuncture. [file 1741-7015-8-75-S1.DOC]

**Additional File 1**

**Search strategy Medline 19.04.10 (1966-2010 week 15)**

| [**# ▲**](http://ovidsp.tx.ovid.com/sp-2.3.1b/ovidweb.cgi?&S=DOLPFPMIHFDDHDLJNCELLEJLIHHFAA00&Sort+Sets=descending) | **Searches** | **Results** |
| --- | --- | --- |
| 1 | randomized controlled trial.pt. | 288341 |
| 2 | controlled clinical trial.pt. | 81073 |
| 3 | randomized.ab. | 196238 |
| 4 | placebo.ab. | 118006 |
| 5 | placebo.ab. | 118006 |
| 6 | clinical trials as topic.sh. | 147767 |
| 7 | randomly.ab. | 142606 |
| 8 | trial.ti. | 84864 |
| 9 | 1 or 2 or 3 or 4 or 5 or 6 or 7 | 643642 |
| 10 | exp animals/ not humans.sh. | 3458216 |
| 11 | 9 not 10 | 595418 |
| 12 | acupuncture/ | 885 |
| 13 | exp Acupuncture Therapy/ | 12156 |
| 14 | (acupunct* or electroacupunct* or electro-acupunct* or needl*).mp. | 105212 |
| 15 | (PLACEBO* or MOCK* or SHAM* or FAKE* or VEHICLE* or DUMM* or ATTENTION* CONTROL* or PSEUDO* TREAT* or MINIMAL ACUPUNCT* or non-Penetrating or NO ACUPUNCT* or NO?Acupunct* or non-specific or non?specific or single-blind*).mp. | 351006 |
| 16 | 12 or 13 or 14 | 105463 |
| 17 | 16 and 15 and 11 | 1610 |
| 18 | limit 17 to "review articles" | 252 |
| 19 | 17 not 18 | 1358 |
| 20 | limit 19 to chinese | 54 |
| 21 | 19 not 20 | 1304 |

**Search strategy Embase search**, 19.04.10 (1988-2010 week 15)

| [**# ▲**](http://ovidsp.tx.ovid.com/sp-2.3.1b/ovidweb.cgi?&S=GLFJFPMJJCDDDDPANCELNDGCMEBGAA00&Sort+Sets=descending) | **Searches** | **Results** |
| --- | --- | --- |
| 1 | (random$ or placebo$).ti,ab. | 444131 |
| 2 | ((single$ or double$ or triple$ or treble$) and (blind$ or mask$)).ti,ab. | 90034 |
| 3 | controlled clinical trial$.ti,ab. | 10304 |
| 4 | RETRACTED ARTICLE/ | 3344 |
| 5 | or/1-4 | 463656 |
| 6 | (animal$ not human$).sh,hw. | 1592491 |
| 7 | 5 not 6 | 417487 |
| 8 | acupuncture analgesia/ or acupuncture/ | 11687 |
| 9 | acupuncture therapy.mp. | 326 |
| 10 | (acupunct* or electroacupunct* or electro-acupunct* or needl*).mp. | 67272 |
| 11 | 8 or 9 or 10 | 67272 |
| 12 | (PLACEBO* or MOCK* or SHAM* or FAKE* or VEHICLE* or DUMM* or ATTENTION* CONTROL* or PSEUDO* TREAT* or MINIMAL ACUPUNCT* or NON?Penetrating or NO ACUPUNCT* or NO?Acupunct* or non-specific or non?specific or single-blind*).mp. | 321791 |
| 13 | 7 and 11 and 12 | 1624 |
| 14 | limit 13 to chinese | 28 |
| 15 | 13 not 14 | 1596 |
| 16 | review/ | 967463 |
| 17 | 15 not 16 | 1268 |
| 18 | 15 not 16 | 1268 |
| 19 | review*.ti. | 118018 |
| 20 | 18 not 19 | 1225 |

**Table S1 -** Additional publications related to included studies

| First author year | Content and relation to included study |
| --- | --- |
| Aune 1998 [80] | Norwegian publication on Aune 1998 |
| Brinkhaus 2003 [81] | Protocol publication for Brinkhaus 2006 and Witt 2005 |
| Brinkhaus 2006 [82] | Publication of treatment details for Brinkhaus 2006 |
| Brinkhaus 2007 [83] | Publication of treatment details for Witt 2005 |
| Hay 2004 [84] | Protocol publication for Foster 2007 |
| Linde 2006 [85] | Publication of treatment details for Linde 2005 |
| Melchart 2003 [86] | Protocol publication for Melchart 2005 |
| Melchart 2005 [87] | Publication of treatment details for Melchart 2005 |
| Röschke 1998 [88] | German version of Röschke 2000 |
| Smith 2002 [89] | Publication focussing on placebo aspect in Smith 2002 |
| Smith 2002 [90] | Publication reporting on pregnancy outcomes in Smith 2002 |

**Table S2 -** Excluded studies

| First author year | Reason for exclusion |
| --- | --- |
| **Selection criteria not met** | |
| Bier 2002 [91] | No untreated (no acupuncture) control group |
| Bullock 2002 [92] | No data for meta-analysis |
| Chow 1983 [93] | No-treatment control not randomized (cross-over study in induced asthma attacks) |
| Cottraux 1983 and 1986 [94-95] | No sham acupuncture group (only placebo medication) |
| Dundee 1989 [96] | Pooled post-hoc analysis of several separate studies covering the data from the study Dundee et al. 1986 included in the review |
| Fung 1986 [97] | No-treatment control not randomized (cross-over study in induced asthma attacks) |
| Gerardi 1983 [98] | Unclear whether randomised, unclear whether no treatment control included |
| Gosman-Hedström 1998 [99] | Penetrating sham at the correct points (less points, superficial needling) |
| Kaptchuk 2008 [22] | No true acupuncture intervention; first phase of the included trial Lembo et al. 2009 |
| Lin 2002 [100] | Sham group received needling at correct points (intervention group additional electro-stimulation) |
| Lin 2009 [101] | Trial on recovery ability of healthy basketball athletes |
| Ludwig 1999 [102] | Only physiological outcomes reported |
| Rösler 2003 [103] | No data for meta-analysis |
| Sertel 2009 [104] | Different timing of measurements in no acupuncture control group; large baseline differences hardly compatible with randomization |
| Sprott 1993 and 1998 [105-106] | No data for meta-analysis |
| Tashkin 1977 [107] | No-treatment control not randomized (cross-over study in induced asthma attacks) |
| **Study protocols of potentially relevant ongoing trials** | |
| Kim 2009 [108] | Protocol of probably eligible trial |
| Vas 2008 [109] | Protocol of a study for which eligibility could not assessed with certainty |
| **Trials likely to be eligible for which only an abstract with minimal information was available and for which further information could not be obtained** | |
| Benson 2006 [70] | Abstract with minimal information (senior author contacted – no feedback); probably trial meeting inclusion criteria; only dichotomous outcomes reported (n = 200) |
| Fratterelli 2008 [71] | Abstract with minimal information (senior author contacted – no feedback) ; probably trial meeting inclusion criteria; only dichotomous outcomes reported (n = 1000) |
| **“Borderline” studies** |  |
| Avants 2000 [72] | No acupuncture group received minimal relaxation therapy (not provided in other groups) |
| Berman 2004 [73] | No acupuncture group received group intervention plus educational information (not provided in other groups) |
| Margolin 2002 [74] | No acupuncture group group received minimal relaxation therapy (not provided in other groups) |
| Scharf 2006 [75] | No acupuncture group hat additional physician visits and received more anti-inflammatory drugs |
| Shen 2000 [76] | Publication reports asymmetric confidence intervals; unambiguous calculation of a standard deviation for effect size estimation not possible |

**Table S3 -** Subgroup and sensitivity analyses

|  | Sham vs. no acupuncture | | | True vs. sham acupuncture | | |
| --- | --- | --- | --- | --- | --- | --- |
|  | SMD (95% CI)  Random effects | I² | p | SMD (95% CI)  Random effects | I² | p |
| **SUBGOUP ANALYSES** |  |  |  |  |  |  |
| **Sample size**  - larger comparisons (≥ 100 patients, n = 12)  - smaller studies (< 100 patients, n = 20)  Test for subgroup differences | -0.51 (-0.55, -0.36)  -0.38 (-0.58, -0.18) | 63%  47% | <0.001  <0.001  0.29 | -0.15 (-0.31, 0.01)  -0.59 (-0.93, -0.24) | 77%  83% | 0.07  <0.001 |
|  |  |  |  |  |  |  |
| **Risk of bias**  - lower risk of bias (13)  - higher risk of bias (19)  Test for subgroup differences | -0.53 (-0.67; -0.38)  -0.37 (-0.56; -0.18) | 54%  53% | <0.001  <0.001  0.07 | -0.21 (-0.41; -0.01)  -0.54 (-0.85; -0.22) | 81%  84% | 0.04  <0.001  0.04 |
|  |  |  |  |  |  |  |
| **Clearly defined main outcome measure**  - yes (15)  - no (17)  Test for subgroup differences | -0.51 (-0.68, -0.34)  -0.39 (-0.55, -0.24) | 66%  35% | <0.001  <0.001  0.20 | -0.25 (-0.48, -0.03)  -0.51 (-0.79, -0.22) | 82%  84% | 0.03  <0.001  0.28 |
|  |  |  |  |  |  |  |
| **Intensity of cointerventions**  - intense (7)  - less intense (25)  Test for subgroup differences | -0.29 (-0.47; -0.11)  -0.49 (-0.63; - 0.36) | 21%  52% | 0.002  <0.001  0.002 | -0.07 (-0.29; 0.16)  -0.47 (-0.68; -0.25) | 44%  86% | 0.58  <0.001  <0.001 |
|  |  |  |  |  |  |  |
| **Skin penetration in sham group**  - yes (27)  - no (5)  Test for subgroup differences | -0.40 (-0.52; -0.28)  -0.71 (-1.04; -0.38) | 41%  77% | <0.001  <0.001  0.02 | -0.37 (-0.55; -0.18)  -0.38 (-0.94; 0.19) | 78%  93% | <0.001  0.19  0.07 |
|  |  |  |  |  |  |  |
| **Skin penetration/point selection sham**  - yes – non-indicated points (6)  - yes – outside points (21)  - no – at correct points (3)  - no – outside points (2)  Test for subgroup differences | -0.48 (-0.90, -0.06)  -0.41 (-0.53, -0.30)  -0.59 (-1.00, -0.18)  -0.98 (-1.29, -0.67) | 71%  25%  82%  0% | 0.03  <0.001  0.004  <0.001  0.008 | -0.32 (-0.83, 0.18)  -0.38 (-0.58, -0.18)  -0.51 (-1.40, 0.38)  -0.25 (-0.53, 0.04) | 82%  78%  97%  0% | 0.21  <0.001  0.26  0.09  0.16 |
|  |  |  |  |  |  |  |
| **Consent analysis**  - sham described as other treatment (12)  - sham described as placebo (3)  Test for subgroup differences  - consent procedure not reported (17) | -0.57 (-0.74, -0.41)  -0.65 (-1.15, -0.16)  -0.31 (-0.46, -0.15) | 55%  58%  33% | <0.001  0.01  0.14  <0.001 | -0.15 (-0.34, 0.04)  -0.34 (-1.18, 0.50)  -0.58 (-0.91, -0.26) | 76%  84%  85% | 0.10  0.22  0.52  <0.001 |
|  |  |  |  |  |  |  |
| **SENSITVITY ANALYSES** |  |  |  |  |  |  |
| **Inclusion of borderline studies**  - chronic pain studies (15)  - short term studies (6)  - other studies (16)  Test for subgroup differences  Total (37) | -0.49 (-0.62; -0.36)  -0.23 (-0.50; 0.04)  -0.37 (-0.56; -0.18)  -0.42 (-0.52; -0.31) | 50%  0%  63%  53% | <0.001  0.09  <0.001  0.12  <0.001 | -0.39 (-0.60; -0.19)  -0.34 (-0.79; -0.12)  -0.31 (-0.57; -0.04)  -0.35 (-0.50; -0.20) | 86%  64%  81%  82% | <0.001  0.15  0.02  <0.001  <0.001 |
|  |  |  |  |  |  |  |
| **Outcomes sensitivity analysis**  - chronic pain studies (14)  - short term studies (6)  - other studies (12)  Test for subgroup differences  Total (32) | -0.54 (-0.67, -0.40)  -0.16 (-0.72, 0.39)  -0.39 (-0.65, -0.14)  -0.43 (-0.56, -0.29) | 44%  73%  71%  66% | <0.001  0.56  0.003  0.004  <0.001 | -0.47 (-0.72, -0.22)  -0.34 (-0.79, 0.12)  -0.28 (-0.59, 0.03)  -0.37 (-0.55, -0..20) | 86%  64%  82%  82% | <0.001  0.15  0.07  0.30  <0.001 |
|  | RR (95% CI) |  |  | RR (95%CI) |  |  |
| **Dichotomous outcomes**  - chronic pain studies (10)  - short term studies (4)  - other studies (9)  Test for subgroup differences  Total (24) | 0.75 (0.68; 0.83)  0.92 (0.81; 1.06)  0.87 (0.64; 1.18)  0.80 (0.73; 0.88) | 47%  0%  68%  54% | <0.001  0.24  0.38  0.02  <0.001 | 0.72 (0.58; 0.90)  0.51 (0.32; 0.81)  1.09 (0.88; 1.36)  0.77 (0.64; 0.92) | 71%  36%  37%  72% | 0.003  0.005  0.43  <0.001  0.004 |

SMD = standardized mean difference, RR = relative risk, 95% CI = 95% confidence interval; I² = index for heterogeneity in per cent, p = p-value for a significant difference between groups

**Figure S1 - The “specific” effect of acupuncture (difference between groups receiving acupuncture and sham acupuncture)**

SD = standard deviation; Total = number of patients; 95% CI = 95% confidence interval; IV = inverse variance method; Random = random effects model; df = degrees of freedom

**Figure S2 - Funnel plot of studies comparing acupuncture vs. sham acupuncture**

SE = standard error; SMD = standardized mean difference

**Figure S3 - The “total” effect of acupuncture (difference between groups receiving acupuncture and no acupuncture)**

SD = standard deviation; Total = number of patients; 95% CI = 95% confidence interval; IV = inverse variance method; Random = random effects model; df = degrees of freedom

**Figure S4 - Funnel plot of studies comparing acupuncture vs. no acupuncture**

SE = standard error; SMD = standardized mean difference
